# Nitrogen in plants: from nutrition to the modulation of abiotic stress adaptation

**DOI:** 10.1007/s44154-021-00030-1

**Published:** 2022-01-07

**Authors:** Jia Yuan Ye, Wen Hao Tian, Chong Wei Jin

**Affiliations:** 1 State Key Laboratory of Plant Physiology and Biochemistry, College of Natural Resources and Environmental Science, Zhejiang University, Hangzhou, 310058 China; 2 State Key Laboratory of Rice Biology, China National Rice Research Institute, Zhejiang, 310006 Hangzhou China

**Keywords:** Nitrate, Ammonium, Uptake, Signaling, Abiotic stress

## Abstract

Nitrogen is one of the most important nutrient for plant growth and development; it is strongly associated with a variety of abiotic stress responses. As sessile organisms, plants have evolved to develop efficient strategies to manage N to support growth when exposed to a diverse range of stressors. This review summarizes the recent progress in the field of plant nitrate (NO_3_^-^) and ammonium (NH_4_^+^) uptake, which are the two major forms of N that are absorbed by plants. We explore the intricate relationship between NO_3_^-^/NH_4_^+^ and abiotic stress responses in plants, focusing on stresses from nutrient deficiencies, unfavorable pH, ions, and drought. Although many molecular details remain unclear, research has revealed a number of core signaling regulators that are associated with N-mediated abiotic stress responses. An in-depth understanding and exploration of the molecular processes that underpin the interactions between N and abiotic stresses is useful in the design of effective strategies to improve crop growth, development, and productivity.

## Introduction

Nitrogen is an essential macronutrient for plants, where its availability is a determinant of plant productivity (Chen et al. 2020). Nitrate (NO_3_^-^) and ammonium (NH_4_^+^) are the two major forms of N that are absorbed by plants; however, both forms are in short supply in agricultural and natural ecosystems (Crawford and Forde 2002). To achieve sufficient crop production levels and satisfy the global food demands, more than 110 Tg of N fertilizer is applied annually to crops; as such, the global demand for agricultural N fertilizer continues to escalate (Schroeder et al. 2013). However, the excessive input of N fertilizer and the inappropriate application of fertilization methods results in low N use efficiency (NUE), where 50–70% of the applied N fertilizer is lost to the surrounding environment, causing serious environmental problems, such as soil acidification and the eutrophication of water (Guo et al. 2010; McAllister et al. 2012; Kissel et al. 2020).

Plants live in dynamic and complex environments that often contain sources of stress (Zhu 2016). As plants are sessile organisms, they are unable to select their growth environment, and are limited to adapting to such environments. While N is an essential macronutrient for plant growth and development, it is also closely associated with plant adaptations to various abiotic stressors. For example, the competition or coordination between NO_3_^-^/NH_4_^+^ and other ions across the plasmalemma, affects plant resilience to stressors such as salt, potassium deficiency, and heavy metal toxicity; plants with low resilience require more fertilizer compared with plants with high resilience (Zhu 2016). As N is considered the most important nutrient for plant growth from a quantitative perspective, plants have evolved efficient strategies to manage N levels in response to various complex stressors (Nacry et al. 2013). As such, understanding the interactions between N and abiotic stress in plants is crucial to optimize the use of N fertilizers, while keeping the balance between application and the adverse effects of abiotic stresses. This understanding is important for improving modern agricultural systems and developing sustainable agricultural practices. This review briefly summarizes the process of NO_3_^-^/NH_4_^+^ uptake in plants and discusses the roles of these two forms of N in relation to different abiotic stressors, including other nutrient deficiencies, unfavorable pH, ionic stress, and drought.

## Nitrogen uptake

### Molecular basis of nitrate uptake

Plants have developed two NO_3_^-^ uptake systems to better adapt to the fluctuating availability of NO_3_^-^ in soils: a high-affinity transport system (HATS) acting at low external NO_3_^-^ levels, while a low-affinity transport system (LATS) operating at high NO_3_^-^ levels (Crawford and Glass 1998; Forde 2000; Lejay and Gojon 2018). In Arabidopsis, two families of transporters, the nitrate transporter 1 or peptide transporter (NRT1/PTR/NPF) and nitrate transporter 2 (NRT2), play a role in root NO_3_^-^ uptake (Wang et al. 2018).

In Arabidopsis plants, NRT1.1 (also known as CHL1 or NPF6.3), was the first transporter that was identified in root NO_3_^-^ uptake and is responsible for most low-affinity NO_3_^-^ uptake in NO_3_^-^-sufficient growth conditions (Tsay et al. 1993; Huang et al. 1996). Subsequent studies have reported that > 75% of the high-affinity NO_3_^-^ uptake in plants was also contributed by NRT1.1 (Wang et al. 1998; Liu et al. 1999). In contrast, recent studies questioned this contribution, as reduced HATS influxes were not observed in the *nrt1.1* mutant under low NO_3_^-^ conditions (compared to wild-type plants) (Touraine and Glass 1997; Muños et al. 2004; Remans et al. 2006). These contradicting findings have obfuscated the role of NRT1.1 in root NO_3_^-^ uptake. Recently, Ye et al. (2019) clarified that the critical factor in these contradictory conclusions was the varying extent of interference in NO_3_^-^ uptake by NRT2.1 and NRT2.2. An *nrt1.1/2.1/2.2* triple deletion mutant was generated to evaluate the role of NRT1.1 in high-affinity NO_3_^-^ uptake. The difference in NO_3_^-^ uptake between the *nrt1.1/2.1/2.2* and *nrt2.1/2.2* mutants showed that NRT1.1 contributed to ~ 12% of the high-affinity NO_3_^-^ uptake in Arabidopsis (Ye et al. 2019). The switch from the low-affinity to high-affinity mode of NRT1.1 was regulated by the phosphorylation of NRT1.1 on the T101 residue (Liu and Tsay 2003). Ho et al. (2009) found that the calcineurin B-like interacting protein kinase, CIPK23, was responsible for phosphorylation in response to low NO_3_^-^ cues, in which the process required the action of CBL9. NRT1.2 is another NRT1 transporter is expressed in epidermal cells and root hairs; it also absorbs NO_3_^-^ from soils, though it is only directly involved in constitutive low-affinity NO_3_^-^ uptake (Huang et al. 1999).

**Fig. 1:**
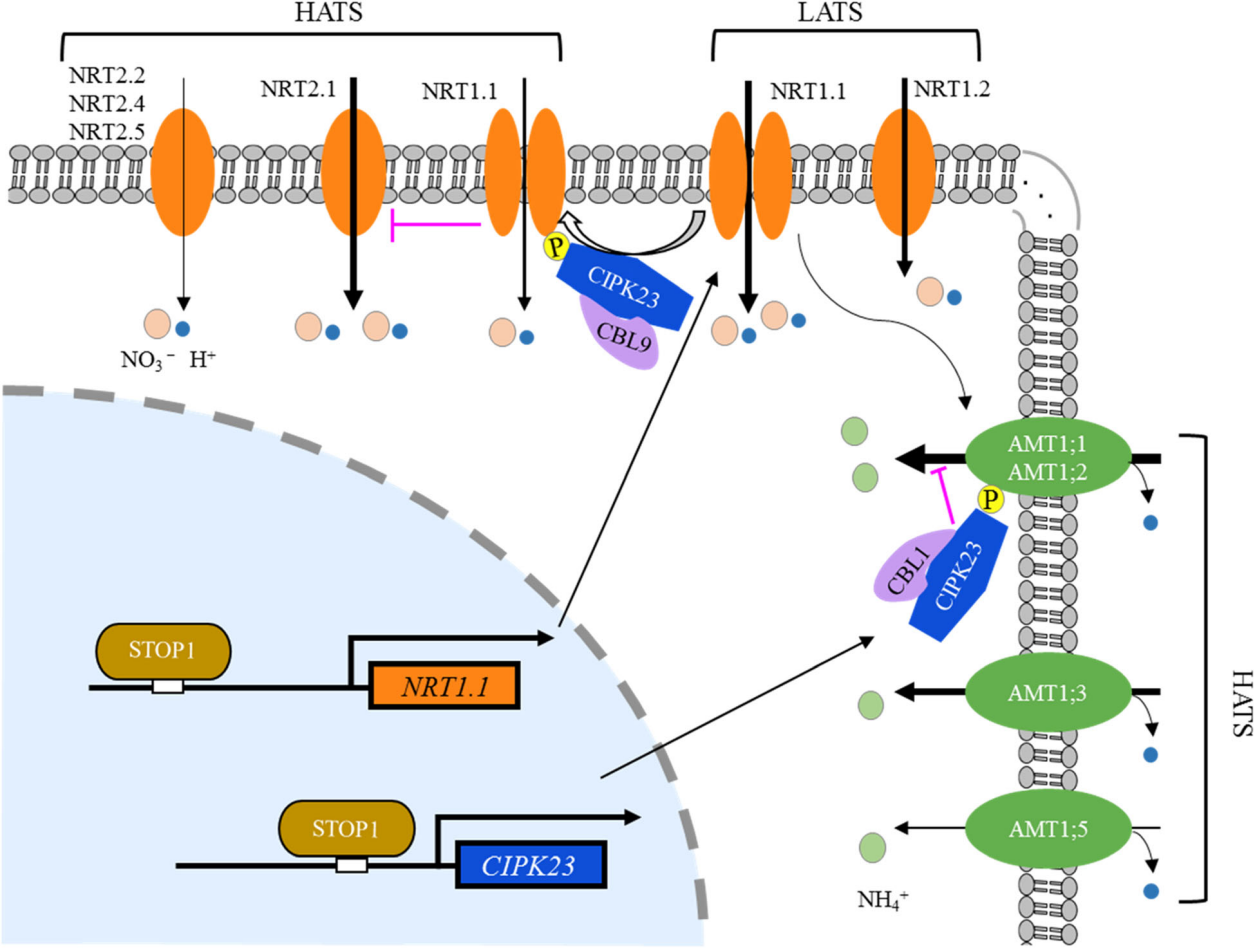
Schematic of membrane transporters involved in the root uptake of nitrate and ammonium in *Arabidopsis thaliana*. The diagram represents an idealized root cell, disregarding developmental differentiation. Major signaling pathways regulating the expression and biochemical activity of these transporters are also included. Proteins are grouped as high-affinity transport systems (HATS) or lowaffinity transport systems (LATS), based on their affinity for the substrate. The contribution of transporters to N uptake is illustrated by the width of the solid lines. NRT1.1 are LATS and HATS, depending on phosphorylation by the CIPK23-CBL complex. The magenta lines indicate negative regulation

As opposed to NRT1 transporters, all NRT2 genes encode for high-affinity NO_3_^-^ transporters, including NRT2.1, NRT2.2, NRT2.4, and NRT2.5, which are expressed in the roots of plants (Wang et al. 2018; Fig. 1). Among these genes, *NRT2.1* is the major contributor to high-affinity NO_3_^-^ uptake, and its disruption reduces HATS activity levels by up to 72% (Li et al. 2007). NRT2.2 exhibits similar expression patterns and properties to those of NRT2.1 (Li et al. 2007); however, its disruption in the *nrt2.1* mutant only reduces HATS activity levels by 8% (Li et al. 2007), suggesting that there is only a marginal contribution by NRT2.2 to HATS. Two other NRT2 transporters, NRT2.4 and NRT2.5, were expressed only in response to extreme N starvation, making only minor contributions to NO_3_^-^ uptake (Kiba et al. 2012; Lezhneva et al. 2014; Liu et al. 2020a).

### Molecular basis of ammonium uptake

The Arabidopsis genome has six ammonium transporters (*AMT*), all of which encode high-affinity NH_4_^+^ transporters and their expression is upregulated under N limitation (Yuan et al. 2007). Among these six genes, *AMT1;4* is expressed in the shoots, while the other five genes (i.e., *AMT1;1*, *AMT1;2*, *AMT1;3*, *AMT1;5,* and *AMT2;1*), are expressed in the roots. To date, there has been no evidence of the contribution of AMT2;1 to high-affinity NH_4_^+^ influx (Sohlenkamp et al. 2002; Yuan et al. 2007). However, under NH_4_^+^ supply, AMT2;1 is mainly expressed in the pericycle and may contribute to the root-to-shoot translocation of NH_4_^+^ (Giehl et al. 2017); as such, NH_4_^+^ uptake is largely mediated by other AMT1s. *AMT1;1*, *AMT1;3*, and *AMT1;5* are mainly expressed in the root tips and epidermal cells to uptake NH_4_^+^ from the soil (Loqué et al. 2006; Yuan et al. 2007), whereas *AMT1;2* is localized in the endodermis and cortex to transport apoplastic NH_4_^+^ into the cell (Neuhäuser et al. 2007). Studies on NH_4_^+^ influx in Arabidopsis mutants have shown that AMT1;1, AMT1;2, and AMT1;3, collectively contribute to ~ 90% of the overall high-affinity NH_4_^+^ uptake capacity, while AMT1;5 mediates the remaining capacity (Loqué et al. 2006; Yuan et al. 2007). These results confirm that AMT1;1*,* AMT1;2*,* and AMT1;3 are major contributors to high-affinity NH_4_^+^ uptake, demonstrating that plants utilize different NH_4_^+^ transporters for effective NH_4_^+^ uptake under low N availability.

As high NH_4_^+^ concentrations are toxic, AMTs in Arabidopsis are efficiently deactivated by phosphorylation to prevent toxicity under high NH_4_^+^ availability (Lanquar et al. 2009). Neuhäuser et al. (2007) demonstrated that external NH_4_^+^ promotes the phosphorylation of a conserved threonine residue in the cytosolic C-terminal domain of AMT1 proteins. Subsequently, Straub et al. (2017) found that CIPK23 physically interacted with and phosphorylated AMT1;1 and AMT1;2. Moreover, they reported that the inhibiting effect of CIPK23 on AMT1 activity was CBL1-dependent and CBL9-independent (Straub et al. 2017). This is contrary to NRT1.1, which was found to be phosphorylated by CIPK23, in a CBL9-dependent manner (Ho et al. 2009); the CBL-mediated specificity may be attributable to this difference.

## Roles of nitrogen in plant adaptation to nutrient deficiency

### Phosphate deficiency

Phosphorus (P) is another essential macronutrient required for plant growth. Several studies have shown that the N and P uptake processes interact with each other, and require coordination to achieve optimal growth and nutritional balance in an environment with fluctuating nutrient availability (Gusewell 2004; Kant et al. 2011; Hu and Chu 2020). In most cases, N uptake in various plant species reduces under phosphate (Pi) deficiency when compared to Pi sufficiency (Lee 1982; Rufty et al. 1990; Wang et al. 2020); this is most likely to maintain the balance between N and P (Ueda et al. 2020).

In recent years, interaction mechanisms between N and P, particularly NO_3_^-^ and Pi, have been studied extensively in Arabidopsis and rice. A previous study showed that nitrogen limitation adaptation (*NLA*) and micro-RNA827 were involved in maintaining NO_3_^-^-dependent Pi homeostasis in Arabidopsis (Kant et al. 2011). NO_3_^-^-inducible GARP-type transcriptional repressor 1 (NIGT1) proteins were initially identified as mediators of NO_3_^-^ responses in rice (Sawaki et al. 2013); subsequently, NIGT1/HRS1 was found to integrate N and P signals in Arabidopsis (Medici et al. 2015). The expression of *NIGT1* was induced by NO_3_^-^ supply in an NRT1.1-dependent manner and was inhibited by Pi deficiency (Medici et al. 2015). NIGT1 was also found to repress NO_3_^-^ uptake in response to Pi deficiency by directly modulating *NRT2.1* and *NRT2.4* expression (Kiba et al. 2018; Maeda et al. 2018). Furthermore, AtNIGT1 proteins modulate Pi starvation signaling and uptake by directly repressing the expression of *SPX* (Ueda et al. 2020); this inhibits the master regulator, phosphate starvation response 1 (*PHR1*), in response to Pi starvation (Rubio et al. 2001). A recent report has shown that NIGT1.2 can directly downregulate the transcription of the NO_3_^-^ transporter gene *NRT1.1* and upregulate the expression of the *phosphate transporter1;1* (*PHT1;1*) and *phosphate transporter1;4* (*PHT1;4*) Pi transporter genes by binding to their promoters; this will promote Pi uptake and inhibits NO_3_^-^ influx during Pi deficiency (Wang et al. 2020). In rice plants, OsNRT1.1B, which is a functional homolog of AtNRT1.1, also modulates optimal NO_3_^-^-phosphate acquisition (Hu et al. 2019). The repressor protein, OsSPX4, is able to interact with OsNLP3 and OsPHR2 to inhibit the NO_3_^-^ and Pi starvation responses, respectively (Hu et al. 2019). Interestingly, OsNRT1.1B uses the plasma membrane-localized E3 ubiquitin ligase, NBIP1 and OsSPX4, to form a complex that promotes OsSPX4 ubiquitination and degradation in an NO_3_^-^-dependent manner (Hu et al. 2019). Thus, OsNLP3 and OsPHR2 may be released and translocated to the nucleus, transducing N and P signals (Hu et al. 2019).

Unlike NO_3_^-^, the application of NH_4_^+^ fertilizers has been known to improve soil Pi uptake in agriculture (Thomson et al. 1993); however, the underlying basis linking NH_4_^+^ and Pi signals remains unclear. Recently, the transcription factor, sensitive to proton rhizotoxicity 1 (STOP1), has been found to coordinate NH_4_^+^ and Pi acquisition in Arabidopsis (Tian et al. 2021). NH_4_^+^ uptake mediated by AMTs induces rapid acidification in the rhizosphere in response to Pi deficiency. This triggers the accumulation of STOP1 in the nucleus and the subsequent excretion of organic acids by the cell, which helps to solubilize P from insoluble Pi sources (Tian et al. 2021). Interestingly, NH_4_^+^ absorption was downregulated by the protein kinase, CIPK23, whose expression was directly controlled by STOP1 when NH_4_^+^ reached toxic levels (Tian et al. 2021). Collectively, Tian et al. (2021) demonstrates that STOP1 plays a key role in coordinating NH_4_^+^ and P signals. The next challenge is determining how plants detect fluctuating environmental conditions to activate STOP1 accumulation and trigger the associated molecular and physiological responses.

### Potassium deficiency

Potassium is another essential macronutrient for plant growth and development, alongside N and P. The absorption and translocation of K^+^ and NO_3_^-^ are positively correlated in plants (Blevins et al. 1978; Triplett et al. 1980; Coskun et al. 2017; Li et al. 2017); the presence of K^+^ increases NO_3_^-^ uptake and assimilation in wheat seedlings (Blevins et al. 1978), in turn, NO_3_^-^ promotes K^+^ uptake and root-to-shoot translocation (Triplett et al. 1980). Recently, Fang et al. (2020) showed that NRT1.1 was upregulated at the transcriptional and post-transcriptional levels in response to low-K stress. They demonstrated that NO_3_^-^ uptake by NRT1.1 in the root epidermis-cortex, favored K^+^ uptake, playing an important role in improving plant tolerance to low-K stress. The uptake of K^+^ across the plasmalemma of the root cortex cells was coupled with proton (H^+^) efflux mediated by H^+^-ATPase (Zhang et al. 2017). The optimum pH for plasmalemma H^+^-ATPase activity in plant roots was found to be ∼6.2–6.5 (Cowan et al. 1993; Zhu et al. 2009); lowering the pH of growth medium markedly reduced root K^+^ uptake (Fang et al. 2020). The NRT1.1-mediated NO_3_^-^ uptake by the cell was accompanied by the co-transport of extracellular H^+^, which alkalizes the rhizosphere (Marschner 1995; Fang et al. 2016). Thus, the NRT1.1-mediated H^+^/NO_3_^-^ symport of epidermis-cortex cells reduces K^+^ uptake-coupled H^+^ efflux, maintaining a suitable pH in the rhizosphere to optimize H^+^-ATPase activity for K^+^ uptake transporters (e.g., AKT1, HAK5, and KUP7), and enhance root K^+^ uptake (Fang et al. 2020). However, it remains unclear how NRT1.1 is regulated in response to low-K^+^ stress. The process described above is likely to be a general (as opposed to specific), mechanism to regulate NRT1.1 during K^+^ uptake; it also plays a role in the root uptake of similar ions coupled to the H^+^ efflux/influx.

In addition to K uptake, root-to-shoot K translocation is regulated by NRTs; NRT1.5 is a low-affinity NO_3_^-^ transporter that has been identified as a major component involved in this process (Lin et al. 2008). The *nrt1.5* mutants presented disturbed root-to-shoot K allocation (Drechsler et al. 2015; Li et al. 2017). Further investigations showed that while NRT1.5 is a NO_3_^-^ transporter, it can also be an H^+^/K^+^ antiporter; NRT1.5-mediated K^+^ transportation into the xylem is independent of NO_3_^-^ transport (Li et al. 2017; Du et al. 2019). In addition to its expression in the root epidermis-cortex, NRT1.1 is also expressed in the root central vasculature, where it plays a role in the coordination of K^+^/NO_3_^-^ translocation (Fang et al. 2020). However, unlike NRT1.5, NRT1.1 is unable to directly transport K^+^ and its improved K^+^ translocation in the central vasculature is also dependent on pH regulation.

### Iron deficiency

Iron is an essential micronutrient for plant growth and development. The bioavailable Fe in soils, particularly calcareous soils, often fails to meet plant needs, resulting in Fe deficiency and reduced crop yields (Guerinot and Yi 1994; Rodríguez-Celma et al. 2019). In agriculture, the application of NO_3_^-^-N fertilizers often aggravates symptoms of chlorosis induced by Fe deficiency (Zhao and Ling 2007). This may be attribute to the inhibition of Fe^3+^-chelated reductase activity in roots by NO_3_^-^ supply (Nikolic et al. 2007). As previously discussed, cellular NO_3_^-^ uptake is coupled with extracellular H^+^ influx to alkalize the rhizosphere (Marschner 1995; Fang et al. 2016). The alkalized rhizosphere may directly restrict cellular Fe uptake and translocation, reducing Fe accumulation in young leaves. Additionally, the loss of the NRT1.1 function enhances plant tolerance to Fe deficiency (Liu et al. 2015), confirming the negative effect of NO_3_^-^ on Fe nutrition in plants. However, the total Fe accumulation in *nrt1.1* mutants plants was reduced along with lower expression levels of Fe-acquisition genes (e.g., *IRT1*, *FRO2*, and *FIT*) in response to Fe deficiency (Muños et al. 2004; Mao et al. 2014; Liu et al. 2015). These results suggest that NRT1.1-regulated Fe deficiency responses may not be associated with reduced Fe uptake, and may instead be relating to an impaired FIT-dependent Fe deficiency signaling pathway. Regardless, it is still difficult to determine the specific role of NRT1.1-mediated NO_3_^-^ uptake in the Fe deficiency response because of the pleiotropic functions of NRT1.1. One possible explanation may be that NRT1.1 indirectly stimulates Fe depletion during NO_3_^-^ assimilation in plants, as Fe is required as a metal cofactor in the NR assimilation pathway and NR activity increases in the *nrt1.1* mutant under Fe-deficient conditions (Liu et al. 2015); there is still little clarity as to how *nrt1.1* mutants increase NR activity.

In contrast, NH_4_^+^ supply has been reported to promote Fe uptake, as NH_4_^+^ uptake induces H^+^ release from the cell and acidifies the rhizosphere (Mengel and Geurtzen 1988; Kosegarten et al. 1999). Recently, Coleto et al. (2021) reported that the uptake of excess NH_4_^+^ by roots also affected Fe homeostasis in Arabidopsis through an unknown mechanism. This was based on the observed altered gene expression in response to Fe uptake and deficiency under high NH_4_^+^ supply relative to NO_3_^-^ supply. If this impact exists, the effect of NH_4_^+^ on Fe homeostasis may be partially independent of pH regulation; however, further research is required to support this hypothesis.

Notably, some studies have found that Fe concentrations in chlorotic leaves are equal to or (in some cases), greater than those in green leaves (Kosegarten et al. 1999; López-Millán et al. 2000). This suggests that other than the restriction of Fe acquisition by roots, there may be other mechanisms that play in NO_3_^-^-related Fe deficiency chlorosis. To date, various studies have shown that the chlorosis-inducing effect of NO_3_^-^ may be associated with the inactivation of physiological Fe in leaf apoplasts, as NO_3_^-^ results in high apoplastic pH (Hoffmann et al. 1994; Kosegarten and Englisch 1994; Mengel et al. 1994). Additionally, Fe deficiency chlorosis cannot be treated by replacing NO_3_^-^ with NH_4_^+^; in contrast to NO_3_^-^, NH_4_^+^ acidifies leaf apoplasts without any external Fe supply (Aktas and Van Egmond 1979; Mengel and Geurtzen 1988; Kosegarten et al. 1999; López-Millán et al. 2000). Therefore, N-regulated apoplastic pH may play an important role in Fe deficiency responses. Specifically, there may be a central hub that regulates apoplastic pH by modulating the balance between NO_3_^-^ and NH_4_^+^ uptake, in response to Fe deficiency. Further studies are required to test this hypothesis and identify potential candidates involved in these pathways.

### Sulfur and molybdenum homeostasis

Sulfur is an essential constituent of enzymes that participate in N metabolism (Scherer 2008), and S addition increases NUE and biomass in plants (Kaur et al. 2011; Rais et al. 2013; Scherer 2001; Swamy et al. 2005; Carciochi et al. 2020; Salvagiotti et al. 2009; Salvagiotti and Miralles 2008). However, the application of N fertilizer aggravates S deficiency and the extent of this aggravation varies from different forms of N (Clarkson et al., 1989). Although S deficiency reduces NO_3_^-^ uptake and assimilation, it had a reduced impact on NH_4_^+^ uptake (Clarkson et al., 1989). This indicates that NH_4_^+^ may be a better N source for plant growth under S deficiency compared to reduced N supply. Furthermore, De Bona et al. (2011) found that NO_3_^-^ supply increased NO_3_^-^ accumulation and asparagine in plants as a response to S deficiency when compared with NH_4_^+^-N supply as urea, thus repressing nitrate reductase (NR) activity.

Contrary to the positive interactions between N and S, an antagonistic interaction was observed between N and molybdenum (Rietra et al. 2017). Mo acts as catalytic center in NR, and Mo deficiency often leads to N deficiency (Rana et al. 2020). Unlike most elements, Mo bioavailability increases with soil pH (Wichard et al. 2009), and the uptake of N may theoretically regulate plant Mo deficiency responses based on the different effects that various forms of N have on the pH in the rhizosphere. This assumption is supported by the finding that N supply as NH_**4**_^**+**^ decreased the Mo content in cabbage (Domagała-Świątkiewicz and Sady 2012). Further evidence is required to fully test this hypothesis.

### Roles of nitrogen in plant adaptations to H^+^ and alkali stresses

Acidic soils are widespread, spanning approximately half of the global arable land (Kochian et al. 2015). Acidic soils with high H^+^ concentrations are highly toxic, inhibiting plant growth and development (Schubert and Mengel 1990; Iuchi et al. 2007). The H^+^ in acidic soils is also linked to many other stress factors, such as aluminum (Al^3+^) toxicity and Pi deficiency (Sawaki et al. 2009; Kochian et al. 2015). Human activities exacerbate soil acidification, particularly the use of N fertilizers including urea and NH_4_^+^ (Guo et al. 2010; Kissel et al. 2020). The alkalization of the rhizosphere as a result of NO_3_^-^ uptake is critical to counteract H^+^ stress. This conclusion is supported by Fang et al. (2016) who observed an increase in NO_3_^-^ uptake with H^+^ stress through the specific upregulation of NRT1.1 activity; this in turn, alleviated H^+^ stress by increasing the pH in the rhizosphere. By contrast, although H^+^ stress also stimulates the expression of other NRTs, their disruptive function failed to reduce H^+^ stress tolerance (Fang et al. 2016). This may be potentially because NRT1.1 is responsible for the majority of NO_3_^-^ transport (Wang et al. 2018; Fang et al. 2021). Notably, the growth of *nlp7* mutants, which disrupts NO_3_^-^ detection whilst exhibits normal NO_3_^-^ uptake activity levels (Castaings et al. 2009; Marchive et al. 2013), was similar to that of Col-0 plants at low pH (Fang et al. 2016). Furthermore, the growth of *chl1–9* mutants, which disrupts NO_3_^-^ uptake activity but exhibits normal NO_3_^-^ detection (Ho et al. 2009), was considerably lower than that of Col-0 plants and was similar to the NRT1.1-null mutants (Fang et al. 2016). These findings demonstrate that NO_3_^-^ transport activity, as opposed to NO_3_^-^ signaling, stimulates H^+^ resistance.

Recently, Ye et al. (2021) found that the low pH-related spatial expression pattern of *NRT1.1* in Arabidopsis roots requires the action of the C2H2-type transcription factor, STOP1. The *nrt1.1* and *stop1* mutants, and the *nrt1.1 stop1* double mutant, exhibited a similar phenotype that was hypersensitive to low pH. This indicates that STOP1 and NRT1.1 function in the same pathway in H^+^ tolerance. Molecular assays revealed that STOP1 directly activates *NRT1.1* by binding to its promoter, enhancing the NO_3_^-^ uptake of NRT1.1 (Ye et al. 2021). This improves the NUE of plants and creates a favorable pH in the rhizosphere for root growth by decreasing H^+^ concentrations. CIPK23 which regulates the NO_3_^-^ uptake affinity of NRT1.1 via phosphorylation on the T101 residue (Ho et al. 2009), is also a key target gene of STOP1 (Sadhukhan et al. 2019; Tian et al. 2021). Additionally, NH_4_^+^ transport controlled by STOP1-CIPK23 may acidify the rhizosphere when only NH_4_^+^ is supplied (Tian et al. 2021). However, neither NH_4_^+^ nor NO_3_^-^ uptake mediated by STOP1-CIPK23, resulted in significant changes in terms of H^+^ tolerance (Ye et al. 2021). Therefore, the STOP1-NRT1.1 module is likely to serve as the primary mechanism for plant adaptation to acidic environments. Further studies are needed to elucidate how roots avoid excess H^+^ accumulation in the cytoplasm, after stimulating H^+^-coupled NO_3_^-^ uptake by NRT1.1.

Alkalinized soils are widespread across the earth, in which there is > 434 million ha of alkaline soils in the world (Wang et al. 2008) and > 70% of the land in northeast China is alkaline (Kawanabe and Zhu, 1991). Alkali stress may inhibit NO_3_^-^ uptake and assimilation in plants (Yang et al. 2007; Yang et al. 2008; Wang et al. 2011; Wang et al. 2012). Based on physiological and tandem mass tag-based proteomic analyses, Zhao et al. (2019) found that increased N uptake and assimilation promoted plant tolerance to alkali stress, but the underlying mechanism remain unclear. To date, there has been little research on the mechanism underpinning plant adaptation to acidic or alkali stresses. This may be because both forms of stress are consistently accompanies with other unfavorable stresses, such as Al^3+^ toxicity in acidic soils and salt stress in alkaline soils; these are the issues that attract research attention. Indeed, acidic and alkali stresses, as opposed to the accompanying stresses, have been found to have a destructive effect on plants (Yang et al. 2008; Wang et al. 2011; Ye et al. 2021). Therefore, determining the interaction mechanism between N nutrition and unfavorable pH stresses may be hugely significant to improve plant growth under unfavorable pH stresses and helpful in understanding the accompanying stresses.

## Roles of nitrogen in plant adaptations to ionic stress

### Ammonium toxicity

Although NH_4_^+^ is one of the predominant N sources in many natural ecosystems, excess NH_4_^+^ is toxic to plants (von Wirén et al. 2000; Britto and Kronzucker 2002). Compared to plants growing in high-NO_3_^-^ environments, plants growth in under high-NH_4_^+^ conditions exhibit several distinct toxicity symptoms, such as stunted root systems and leaf chlorosis (Britto and Kronzucker 2002; Li et al. 2014). Previous studies have shown that the excretion of H^+^ and general cation uptake suppression are the major contributors to the impaired growth from high NH_4_^+^ concentrations (von Wirén et al. 2000; Li et al. 2014). Interestingly, NH_4_^+^ toxicity symptoms may be reduced through the concurrent presence of small amounts of NO_3_^-^ (Roosta and Schjoerring 2007; Hachiya et al. 2011). The role of NO_3_^-^ in alleviating NH_4_^+^ toxicity is partially attributed to the increase in the pH in the rhizosphere and stimulation of cation uptake during NO_3_^-^ uptake (Hachiya et al. 2011; Hachiya and Noguchi 2011). Surprisingly, the NRT1.1-null mutants in Arabidopsis showed a higher resistance to high NH_4_^+^ than wild-type plants, suggesting that NRT1.1 alleviates NH_4_^+^ toxicity, independent of NO_3_^-^ uptake (Hachiya et al. 2011; Hachiya and Noguchi 2011). Jian et al. (2018) proposed that NH_4_^+^ toxicity is related to the NRT1.1-mediated signaling process as the NRT1.1^P492L^ point mutant *chl1–9* displayed symptoms that were similar to the wild-type plants under high-NH_4_^+^ conditions. Additional experimental data are required to clarify the exact signaling controlled by NRT1.1 in NH_4_^+^ tolerance. Another plausible explanation of the role of NO_3_^-^ in counteracting NH_4_^+^ toxicity is that it inhibits chloride (Cl^-^) uptake via competition between NO_3_^-^ and Cl^-^. The presence of NH_4_^+^ improves Cl^-^ uptake to maintain balanced charge in the roots; this process is significantly inhibited by NO_3_^-^ (Liu et al. 2020). In addition to being an NO_3_^-^ transporter, NRT1.1 also exhibits Cl^-^ permeability in Arabidopsis and the *Xenopus* oocyte system (Wen et al. 2017; Liu et al. 2020). Therefore, the enhanced NH_4_^+^ tolerance of the *nrt1.1* mutant may be associated with their reduced capacity for Cl^-^ uptake under high-NH_4_^+^ conditions when compared to wild-type plants.

### Salt stress

High levels of salt stress negatively influence plant growth and crop productivity. In recent decades, it has been widely acknowledged that the inhibition of nutrient uptake via competition among sodium and other nutritional ions is a major contributor to high salt stress (Tang at el. 2011; Hessini et al. 2013). NO_3_^-^ application has been demonstrated to increase root uptake and xylem loading of Na^+^, increasing salinity-driven root inhibition (Álvarez-Aragón et al. 2016; Álvarez-Aragón and Rodríguez-Navarro 2017). Based on kinetic data of NO_3_^-^-dependent Na^+^ uptake at various Na^+^ concentrations, Álvarez-Aragón and Rodríguez-Navarro (2017) proposed that Na^+^ may be co-transported with NO_3_^-^. Although the co-transport of Na^+^ and NO_3_^-^ has also been described in *Zostera marina* and *Suaeda physophora* (García-Sánchez et al. 2000; Yuan et al. 2010), the transporters that are involved have not yet been identified. Nitrate transporters such as NRT1.1 may be involved in this pathway as Na^+^ is partially deficient in NRT1.1-null mutants only in the presence of NO_3_^-^, when compared to wild-type plants (Álvarez-Aragón and Rodríguez-Navarro 2017).

Several studies have found that NH_4_^+^ exacerbates salt stress more than NO_3_^-^; this is observed only in a limited number of species including pea (*Pisum sativum* L.), poplar (*Populus simonii*), and wheat (*Triticum aestivuni* L.) (Lewis et al. 1989; Frechilla et al. 2001; Meng et al. 2016). In a recent study, Liu et al. (2020) found that when NH_4_^+^ was the sole N source, the loss of the NRT1.1 function improved the salt stress tolerance of plants. Further investigation revealed that excess Cl^-^, as opposed to Na^+^, may be responsible for the hypersensitivity to salt in wild-type Arabidopsis, with NH_4_^+^ as the sole N source (Liu et al. 2020). Consistent with this finding, AtNRT1.1 and its homolog ZmNPF6.4 have Cl^-^ permeability in the *Xenopus* oocyte system; their activity were observed to be considerably inhibited by NO_3_^-^ (Wen et al. 2017). Liu et al. (2020) also showed that the disruptive function of NRT1.1 in *nrt1.1* mutants reduces the transmembrane Cl^-^ influx rate in NH_4_^+^-treated Arabidopsis. Therefore, enhanced Cl^-^ uptake by NRT1.1 in wild-type plants may be a mechanism to induce salt hypersensitivity in plants under high-NH_4_^+^ conditions. Although AtNRT1.1 specifically recognizes NO_3_^-^ and chlorate (ClO_3_^-^) which have similar structures (Parker and Newstead 2014), these results raise the question of how AtNRT1.1 recognizes structurally different substrates of NO_3_^-^ and Cl^-^. Further studies are required to fully elucidate how NRT1.1 balances NO_3_^-^ and Cl^-^ uptake in response to salt stress based on environmental NO_3_^-^ and NH_4_^+^ concentrations.

### Heavy metal stress

Soil heavy metal contamination has become a critical environmental issue because of its adverse ecological effects. Cadmium is one of the most toxic heavy metals in the environment. Studies have shown that NH_4_^+^ application enhances Cd uptake compared to the application of NO_3_^-^; this may be due to a decrease in soil pH (Florjin et al. 1992; Sarwar et al. 2010; Zaccheo et al. 2006). NH_4_^+^-increased Cd uptake may also be associated with NH_4_^+^ interactions with pectate and protein, as well as cell wall polymerization in the roots of *Kandelia obovata* (Chai et al. 2018). By contrast, several other studies have demonstrated that Cd uptake is enhanced by NO_3_^-^ in many species, such as Arabidopsis, rice, potato tubers, and rape (Eriksson 1990; Maier et al. 2002; Hassan et al. 2008; Sarwar et al. 2010). In a hydroponic systems, Xie et al. (2009) found that NO_3_^-^-treated *Thlaspi caerulescens* plants accumulated more Cd than NH_4_^+^-treated plants, despite the pH of the NH_4_^+^ solution being lower. Luo et al. (2012) reported that in pH-buffered hydroponic culture, NO_3_^-^-treated plants accumulate more Cd than NH_4_^+^-treated plants, where the upregulation of Fe uptake was responsible for NO_3_^-^-facilitated Cd accumulation. Within a soil system, Jalloh et al. (2009) showed that rice plants fed NO_3_^-^ had higher Cd concentrations than plants fed NH_4_^+^. These findings indicate that, in addition to changing the pH in the rhizosphere, NO_3_^-^ may regulate Cd uptake in plants, through NO_3_^-^ transporters; this potential has been supported by subsequent evidence. Mao et al. (2014) revealed that in the presence of NO_3_^-^, the functional disruption of NRT1.1 reduces Cd uptake via a synergistic mechanism involving the simultaneous uptake of NO_3_^-^, thus enhancing Cd tolerance. In a recent study, Guan et al. (2021) found that NRT2.1 contributed substantially to facilitate Cd uptake under low-NO_3_^-^ conditions by controlling NO_3_^-^ uptake, further suggests that NO_3_^-^ uptake exacerbates the adverse effects of Cd stress on plants.

In addition to NO_3_^-^ uptake, Cd resistance in plants is also associated with NO_3_^-^ allocation. For example, *NRT1.8*, which removes NO_3_^-^ from xylem vessels, is strongly stimulated by Cd^2+^ stress; the disruption of NRT1.8 increases plant sensitivity to Cd^2+^ stress in an NO_3_^-^-dependent manner (Li et al. 2010). By contrast, *NRT1.5* which transports NO_3_^-^ into the xylem, is strongly downregulated by Cd^2+^ stress; as such, it retains NO_3_^-^ in the roots and contributes to Cd^2+^ tolerance in a similar mechanism to *NRT1.8* (Chen et al. 2012)*.* This demonstrates that plant tolerance to Cd^2+^ stress is regulated by NO_3_^-^ reallocation to roots, mediated by NRT1.8 and NRT1.5 (Chen et al. 2012). This contrasting expression pattern of NRT1.8 and NRT1.5 in response to stress may be a result of crosstalk between ethylene (ET) and jasmonic acid (JA) signaling pathways (Zhang et al. 2014). The NRT1.1-regulated expression of *NRT1.5* and *NRT1.8* in roots may also contribute to Cd^2+^ detoxification (Gojon and Gaymard 2010; Jian et al. 2019). Jian et al. (2019) found that NRG2 operates downstream of NRT1.1 to regulate Cd^2+^/NO_3_^-^ allocation and Cd stress tolerance. Critical factors resulting in the discrepancy described by Mao et al. (2014) and Jian et al. (2019) may be due to the variable NO_3_^-^ and Fe concentrations in the growth medium, as both affect Cd uptake (He et al. 2017).

Zinc (Zn) is an essential nutrient for living organisms, though it may cause phytotoxicity when concentrations exceed requirements. The application of NO_3_^-^ enhances Zn uptake in wheat roots (Erenoglu et al. 2011; Kutman et al. 2011). Additionally, Pan et al. (2020) demonstrated that a disruption in NRT1.1 reduced Zn accumulation in Arabidopsis; as such, the growth of the *nrt1.1* mutant increased under Zn stress, indicating that the NRT1.1-mediated NO_3_^-^ uptake pathway may play an important role in modulating Zn accumulation and tolerance to Zn stress. However, the role of other NRTs in NO_3_^-^-induced Zn accumulation in plants remains unclear. By contrast, NO_3_^-^ decreases Pb uptake in roots and NRT1.1 enhances Pb^2+^ resistance in Arabidopsis (Zhu et al. 2019). Under Pb^2+^ stress, NRT1.1 induces NO_3_^-^ uptake, which decreases the bioavailability of Pb by preventing acidification in the rhizosphere, thus reducing Pb uptake by the roots.

### Roles of nitrogen in plant adaptations to drought stress

Drought stress is a serious threat to plant life and productivity (Ding et al. 2015; Saud et al. 2017). NO_3_^-^ and NH_4_^+^ concentrations have distinct effects on plant performance under drought stress; the application of NH_4_^+^ mitigates the impact of drought on plant growth, while NO_3_^-^ has the opposite effect (Gao et al. 2010; Yang et al. 2012; Ding et al. 2015; Saud et al. 2017). The role of NH_4_^+^ in enhancing the drought tolerance of rice is associated with improved water uptake due to an increase in root numbers and surface area (Li et al. 2009). The decrease in the aerenchyma formation may also contribute to NH_4_^+^-enhanced drought tolerance (Yang et al. 2012). Ding et al. (2015) showed that the increased expression of root aquaporin also contributes to enhanced drought tolerance in rice plants under high-NH_4_^+^ conditions. Currently, there is a lack of evidence that NH_4_^+^ uptake is directly involved in plant drought responses.

The effect of NO_3_^-^ on plant drought responses is associated with NO_3_^-^ transport/assimilation. Under drought stress, many genes involved in NO_3_^-^ transport/assimilation (including *NRT2.5*, *GOGAT*, *GS*, and *AS*), are repressed (Nagy et al. 2013; Singh and Ghosh 2013; Goel and Singh 2015; Duan et al. 2016). The disruptive function of genes responsible for NO_3_^-^ uptake or assimilation pathways improves plant drought response. For example, Guo et al. (2003) found that NRT1.1 which is also highly expressed in guard cells, decreased plant resistance to drought stress. Notably, the reduced stomatal aperture of the Arabidopsis* nrt1.1* mutant was not the result of effects on abscisic acid (ABA) responses, rather, of impaired NO_3_^-^ uptake by guard cells and NO_3_^-^-induced membrane depolarization (Guo et al. 2003). Additionally, mutations in genes encoding NR (*NIA1* and *NIA2*) also exhibited a drought-resistant phenotype; this may be the result of the dual function of smaller mutants and their enhanced sensitivity to ABA (Lozano-Juste and León 2010; Chen et al. 2016). Studies have also reported that the role of NO_3_^-^ in maintaining an open stomata to fix more carbon dioxide (CO_2_) for NO_3_^-^ assimilation may contribute to higher transpiration rates in leaves under drought conditions (Guo et al. 2007; Shi et al. 2014; Ren et al. 2015). Recently, Han et al. (2021) reported that *OsNR1.2* loss-of-function mutants were more tolerant to drought stress than wild-type rice under NO_3_^-^-sufficient conditions, confirming that the suppression of N assimilation contributes to the survival of rice crops under drought stress. Further investigation revealed that the inhibition of the *OsNR1.2* expression and the suppression of N assimilation in response to drought stress is associated with a C_2_H_2_ zinc-finger transcription factor, known as drought and salt tolerance (DST), which plays a role in H_2_O_2_ and cytokinin homeostasis (Huang et al. 2009; Li et al. 2013). Under drought stress, the expression of *DST* is downregulated; this action directly inhibits the activation of DST to its target genes, *OsNR1.2* and *OsPrx24*, thereby facilitate stomata closure via preventing N assimilation and inducing H_2_O_2_ accumulation in the stomatal apparatus, respectively.

In addition to NO_3_^-^ transport and assimilation, NO_3_^-^ signaling also contributes to drought stress tolerance. The disruption of the NIN-like protein 7 (NLP7) in *nlp7* mutants led to the impaired transduction of the NO_3_^-^ signal, resulting in lower transpiration and extended survival under drought stress (Castaings et al. 2009). Many NLP7- and NRT1.1-dependent genes are differentially expressed in response to drought or ABA treatment, suggesting that disruptions in NO_3_^-^ signaling may prompt changes in drought-responsive gene expressions (Araus et al. 2020). These results suggest that NO_3_^-^ plays a role in drought response by regulating the activity of genes involved in NO_3_^-^ uptake/assimilation and signaling, acting through or independent of the ABA pathway.

### Concluding remarks and perspectives

The role of N in various abiotic stress responses has been attracting increasing attention, and there has been considerable progress in understanding these mechanisms. This review explored the effects of NO_3_^-^/NH_4_^+^, particularly NO_3_^-^ uptake, on plant tolerance to different abiotic stressors. These stressors includes nutrient deficiency, unfavorable pH, ionic stress, and drought; the effects of these stressors were investigated in both physiological and molecular terms. Advancing current knowledge on plant regulation of various abiotic stress responses via N is critical to design strategies to improve crop growth, development, and productivity.

**Fig. 2:**
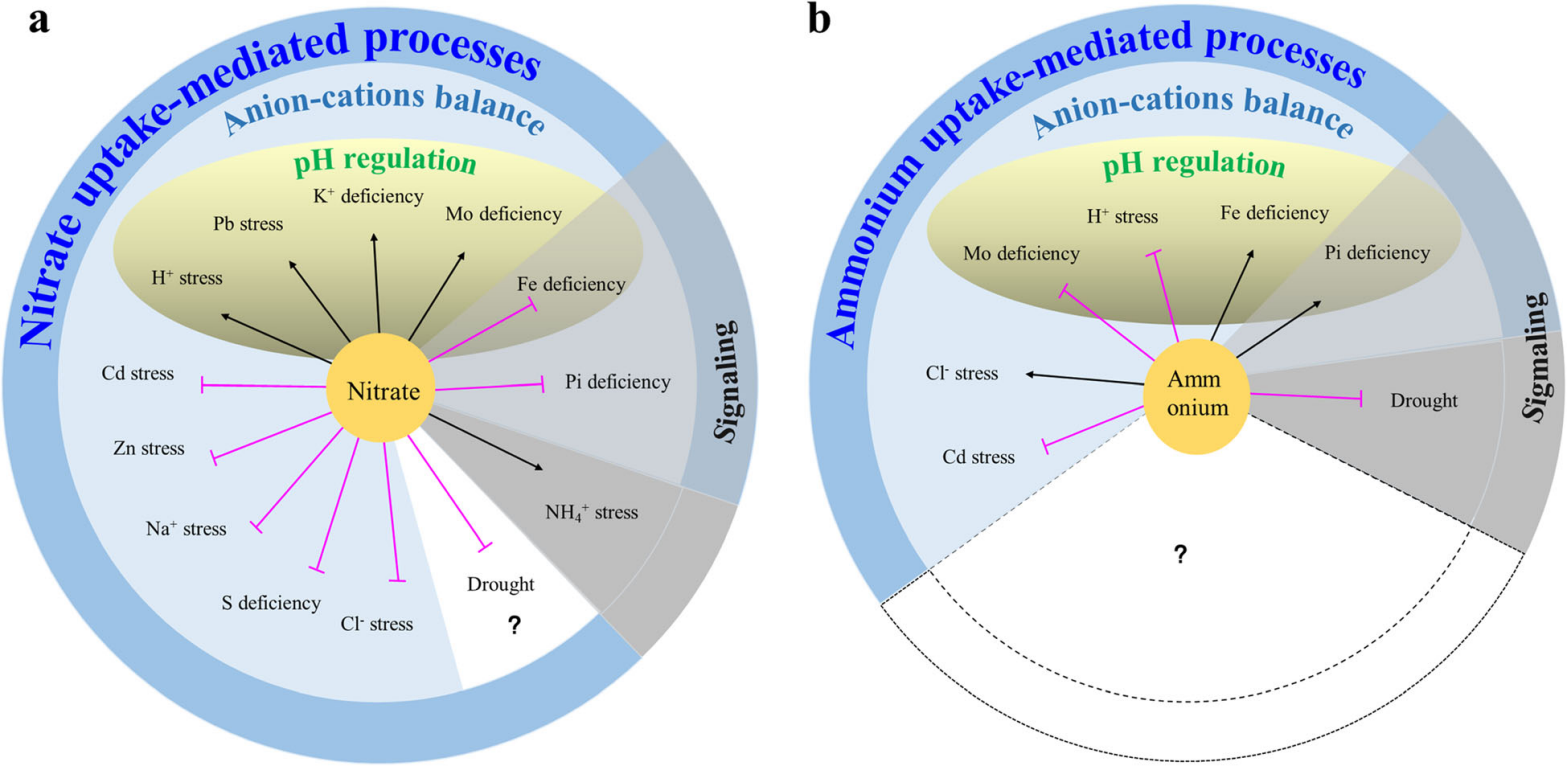
Schematic of the roles of nitrate (NO_3_.^-^)/ammonium (NH_4_.^+^) in plant responses to different stresses: (**a**) NO_3_^-^ uptake plays a role in ~ 90% of the NO_3_^-^-mediated abiotic stress responses, while only NH_4_^ +^ toxicity is mediated by NO_3_^-^ signaling independent to NO_3_^-^ uptake. Pi and Fe deficiency responses were mediated by NO_3_^-^ uptake and NO_3_^-^ signaling. The anion-cation balance mechanism contributes to abiotic stress responses mediated by NO_3_^-^ uptake. NO_3_^-^ uptake facilitates the synergetic transport of cations (e.g., H^+^, K^+^, Na^+^, Cd^2+^, and Zn^2+^), while inhibiting the uptake of anions (e.g., S, Cl^-^, and Pi), acting in 90% of the stress responses mediated by NO_3_^-^. The presence of H^+^/NO_3_^-^ contributes to half of the stresses mediated by the cation-anion balance mechanism, as it effectively increases the pH in the rhizosphere; this affects the bioavailability of many elements. Although NO_3_^-^ uptake exacerbates drought stress, the underlying mechanism remains unclear; (**b**) to date, only seven types of abiotic stresses are mediated by NH_4_^+^, while the role of NH_4_ ^+^ in mediating responses to other types of stress is yet to be identified. Among the known NH_4_ ^+^ -mediated abiotic stress responses, ~ 80% is mediated by NH_4 _^+^ uptake. Plant responses to Pi deficiency and drought stress may require the normal function of NH_4_^ +^ signaling. With the exception of Cl^-^ and Cd^2+^ stresses, the other four NH_4_ ^+^ uptake-mediated abiotic stresses (i.e., H^+^ stress, Pi deficiency, Mo deficiency, and Fe deficiency) were all associated with the antiport of H^+^/NH_4_^+^, which also changes the pH in the rhizosphere. Black arrows demonstrate the positive regulation of stress reduction responses, while the magenta lines indicate negative regulation

As N is always quantitatively required by plants, N uptake may affect the uptake of other ions via a common cation-anion balance mechanism (Narcy et al. 2013; Mao et al. 2014). This competition or coordination mechanism appears to substantially contribute to stress responses mediated by N. For example, NO_3_^-^ uptake facilitates the synergetic transport of cations (such as H^+^, K^+^, Na^+^, Cd^2+^, and Zn^2+^) while it inhibits the uptake of anions (such as Cl^-^ and SO_4_^-^), playing a role in ~ 90% of the stress responses (Fig. 2a). Among these cation-anion balance mechanisms, the presence of H^+^/NO_3_^-^ plays a major role in plant tolerance to stresses as it effectively increases the pH in the rhizosphere, affecting the bioavailability of many elements (Marschner 1995; Fang et al. 2016; Zhu et al. 2019). The H^+^/NH_4_^+^ antiport also changes the pH in the rhizosphere, and may theoretically play a role in many abiotic stress responses (Fig. 2b). To date, only the Pi (Tian et al. 2021) and Fe deficiency responses (Mengel and Geurtzen 1988; Kosegarten et al. 1999) have been associated with the antiport of H^+^/NH_4_^+^, while the role of NH_4_^+^ in other stress responses largely remains unclear. This may be because NH_4_^+^ is toxic and NH_4_^+^ uptake is lower than NO_3_^-^ uptake; further research is required to clarify the role of NH_4_^+^ in plant stress responses. The cation-anion balance mechanism may theoretically depend on the cooperation between anion and cation transporters/channels. However, to date, none of the protein-protein interactions involved in this process have been identified.

Plants are constantly exposed to abiotic stresses under various combinations and their response to one stress may be affected by the presence of other stresses. Thus, plant responses to multiple stresses are not just the simple summations of their responses to each individual stress (Bouain et al. 2019). For example, both Pi or Fe deficiency stress inhibits the growth of primary roots (Gruber et al. 2013; Gutierrez-Alanis et al. 2018), while this effect is eliminated when these stresses are combined as Pi-deficient root elongation is associated with the overaccumulation of Fe (Ward et al. 2008; Müller et al. 2015; Müller et al. 2015; Dong et al. 2017). Similarly, the inhibition of NO_3_^-^ uptake in the *nrt1.1* mutant leads to greater salt stress sensitivity under NO_3_^-^ supply, while it has the opposite effect on salt stress when NH_4_^+^ is the main N source (Álvarez-Aragón et al. 2016; Álvarez-Aragón and Rodríguez-Navarro 2017; Liu et al. 2020b). These examples illustrate how plants respond to combined stress. As N is in short supply in most agricultural and natural systems, it is important to explore plant mechanisms that control growth by integrating and responding to N deficiency signals alongside other stress signals.

Finally, N allocation, distribution, and metabolism may also respond to abiotic stresses. For example, NRT1.5 and NRT1.8 participate in root-to-shoot NO_3_^-^/Na^+^ or NO_3_^-^/Cd transport (Chen et al. 2012). Incorporating these findings may enhance the current understanding of N-modulated abiotic stress responses.

## Data Availability

Not applicable.

## Abbreviations

N: Nitrogen
NO_3_: Nitrate
NH_4_^**+**^: Ammonium
NUE: Nitrogen use efficiency
HATS: high-affinity transport systems
LATS: low-affinity transport systems
NRT1/PTR/NPF: Nitrate Transporter 1/Peptide Transporter
NRT2: Nitrate Transporter 2
NRT: Nitrate Transporter
NR: Nitrate Reductase
AMT: Ammonium Transporter
CBL: Calcineurin B-Like
CIPK23: Calcineurin B-like Interacting Protein Kinase 23
DST: Drought and Salt Tolerance
P: Phosphorus
Pi: Phosphate
NLA: Nitrogen Limitation Adaptation
NIGT1: GARP-type Transcriptional Repressor 1
PHR1: Phosphate Starvation Response 1
PHT1;1: Phosphate Transporter 1;1
PHT1;4: Phosphate Transporter1;4
S: Sulfur
Mo: molybdenum
STOP1: Sensitive To Proton Rhizotoxicity 1
K: Potassium
Fe: Iron
Cl^**-**^: Chloride
ClO_3_^**-**^: Chlorate
H^+^: Proton
Al^3+^: Aluminum
Na^+^: Sodium
NLP7: NIN-like protein 7
Cd: Cadmium
ET: Ethylene
JA: Jasmonic acid
Zn: Zinc
Pb^2+^: Lead
ABA: abscisic acid
